# Do Weather Conditions Still Have an Impact on the COVID-19 Pandemic? An Observation of the Mid-2022 COVID-19 Peak in Taiwan

**DOI:** 10.3390/microorganisms12050947

**Published:** 2024-05-07

**Authors:** Wan-Yi Lin, Hao-Hsuan Lin, Shih-An Chang, Tai-Chi Chen Wang, Juei-Chao Chen, Yu-Sheng Chen

**Affiliations:** 1 Department of Traditional Chinese Medicine, Chang Gung Memorial Hospital, Keelung 204201, Taiwan; wisamur@gmail.com; 2 School of Traditional Chinese Medicine, Chang Gung University, Taoyuan 333323, Taiwan; b0205045@gmail.com (H.-H.L.); anthea91@gmail.com (S.-A.C.); 3 Taiwan Huangdi-Neijing Medical Practice Association (THMPA), Taoyuan 330032, Taiwan; 4 Department of Chinese Acupuncture and Traumatology, Center of Traditional Chinese Medicine, Chang Gung Memorial Hospital, Taoyuan 333008, Taiwan; 5 Department of Atmospheric Sciences, National Central University, Taoyuan 320317, Taiwan; taichirainbow@gmail.com; 6 Department of Statistics and Information Science, Fu Jen Catholic University, New Taipei City 242062, Taiwan; stat1014@mail.fju.edu.tw

**Keywords:** climate factor complex (CFC), COVID-19, Fire-Qi Period, meteorological factors, Taiwan, unease environmental condition factor (UECF), weather

## Abstract

Since the onset of the COVID-19 pandemic in 2019, the role of weather conditions in influencing transmission has been unclear, with results varying across different studies. Given the changes in border policies and the higher vaccination rates compared to earlier conditions, this study aimed to reassess the impact of weather on COVID-19, focusing on local climate effects. We analyzed daily COVID-19 case data and weather factors such as temperature, humidity, wind speed, and a diurnal temperature range from 1 March to 15 August 2022 across six regions in Taiwan. This study found a positive correlation between maximum daily temperature and relative humidity with new COVID-19 cases, whereas wind speed and diurnal temperature range were negatively correlated. Additionally, a significant positive correlation was identified between the unease environmental condition factor (UECF, calculated as RH*Tmax/WS), the kind of Climate Factor Complex (CFC), and confirmed cases. The findings highlight the influence of local weather conditions on COVID-19 transmission, suggesting that such factors can alter environmental comfort and human behavior, thereby affecting disease spread. We also introduced the Fire-Qi Period concept to explain the cyclic climatic variations influencing infectious disease outbreaks globally. This study emphasizes the necessity of considering both local and global climatic effects on infectious diseases.

## 1. Introduction

The emergence of SARS-CoV-2 and the resulting COVID-19 pandemic have had a profound impact worldwide since the first case was reported in Wuhan, China, in December 2019 (WHO 2020) [1,2]. Due to its geographical proximity to China, Taiwan was initially expected to experience a high number of COVID-19 cases, second only to mainland China. However, due to the Taiwanese government’s effective pandemic response strategy, the number of confirmed COVID-19 cases in Taiwan remained relatively low in 2020 [3].

In mid-May 2021, Taiwan experienced a surge in daily confirmed COVID-19 cases due to loosening border control measures and a low vaccination rate. However, the Taiwanese government implemented effective containment measures, resulting in a decrease in daily confirmed cases from as high as 597 on May 28 to as low as 30 cases on 12 July 2021. By October 2021, Taiwan had reported zero indigenous daily cases once again [4], with a first-dose vaccination coverage rate of approximately 65% [5].

**Figure 1:**
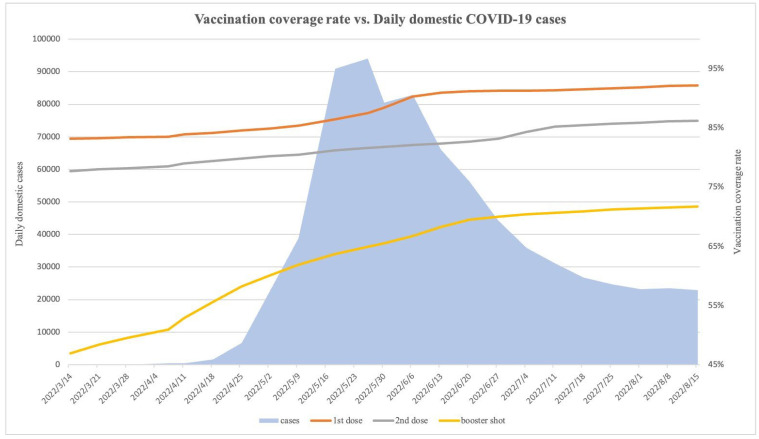
The daily trend in the vaccination coverage rate and the number of COVID-19 cases from 14 March to 15 August 2022 in Taiwan shows that despite high vaccination coverage rates, there was still a resurgence in the number of new COVID-19 cases.

In 2022, Taiwan was expected to enter a stage of coexistence with the epidemic, given its relatively high vaccination coverage rates of 83.9%, 78.9%, and 58% for the first to third doses, respectively [5]. However, a sudden outbreak in late March and early April caused by the SARS-CoV-2 Omicron variant led to a resurgence in the number of new COVID-19 cases despite the high vaccination rate (Figure 1). Therefore, we proposed that weather factors other than health policy and vaccination coverage may still have an impact on the COVID-19 pandemic.

### Literature Review

Since the outbreak of the COVID-19 pandemic, many studies have been attempting to find correlations between weather factors and the spread of the virus. A large study review declared that there was no association between weather conditions and the COVID-19 pandemic [6], but several regional studies have shown a correlation [7,8,9,10]. Research focused on China highlights a positive correlation between temperature and the COVID-19 epidemic [7,11], while other studies suggest that rising temperatures may mitigate the expansion of the pandemic to some extent [12,13]. Ng et al. (2024) provide similar insights for Malaysia, suggesting that regional climatic conditions significantly influence transmission dynamics across the country [14]. Similarly, Saputra et al. (2021) conducted an analysis of climate impacts on the pandemic throughout Asia, emphasizing the complex influence of climate on the spread of COVID-19 [15].

Globally, several studies delve into the broader implications of climate change on COVID-19, revealing complex interactions between various climatic factors and the pandemic [16,17,18,19,20]. Other studies have suggested that the Climate Factor Complex (CFC), a combination of several climate factors, involves a comprehensive consideration of multiple factors [21].

An analysis of the United States’ data from 1 March to 31 October 2020 was conducted, proposing distributed lag non-linear models for the COVID-19 incidence predicted [9]. The peak of the COVID-19 outbreak in 2022 in Taiwan coincided with the Fire Qi period, a concept based on the Yunqi theory of Chinese medicine in Huangdi’s Internal Classic (Huang Di Nei Jing), which is a certain period of the year that is more likely to have infectious disease epidemics. It is usually characterized by unseasonal weather conditions and cyclic climatic variations that can even be observed globally.

Therefore, the present study aimed to investigate the impact of local weather conditions on the daily incidence of COVID-19 in six regions of Taiwan. Additionally, specific weather conditions, such as the unease environmental condition factor (UECF), the Fire-Qi Period, and the cyclic climatic variations affecting the spread of infectious diseases proposed by a prior study [22], were examined.

## 2. Materials and Methods

### 2.1. Study Area

**Figure 2:**
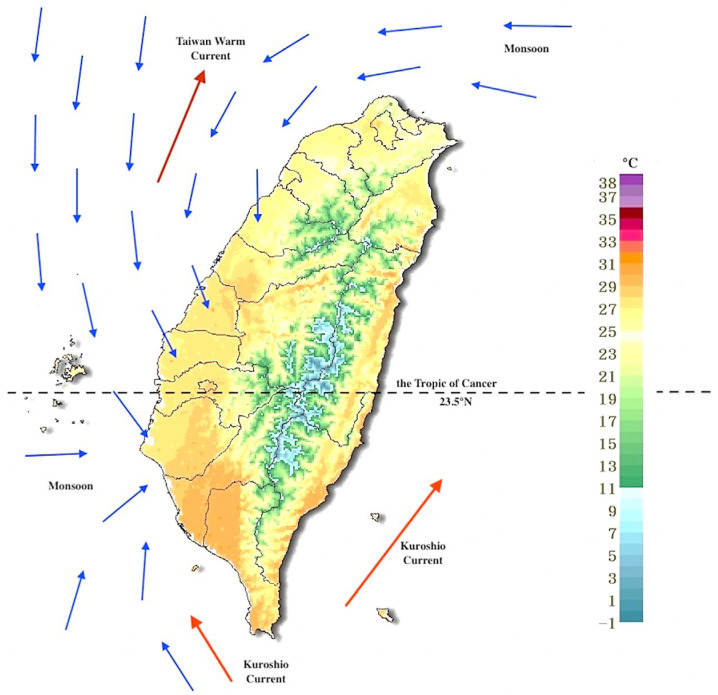
Taiwan is divided by the Tropic of Cancer into tropical and subtropical monsoon climates. Latitude, ocean currents, monsoons, and diverse terrain create varied climatic patterns. The map shows Taiwan’s diverse terrain and temperatures, with blue arrows indicating monsoons and red arrows representing ocean currents. This chart displays temperature patterns in Taiwan during early May, illustrating typical climatic conditions for this period [24].

To analyze the impact of local weather conditions on COVID-19 transmission in Taiwan, this study focused on the island of Taiwan, excluding offshore islands such as Penghu, Kinmen, and Lienchiang. Taiwan is located between Asia, the world’s largest continent, and the Pacific Ocean, the largest ocean. The island is bisected by the Tropic of Cancer (23.5° N), which divides its climate into two distinct zones: a tropical monsoon climate in the south and a subtropical monsoon climate in the north. Characterized by high temperatures and humidity, substantial rainfall, and frequent tropical cyclones during the summer, Taiwan’s climate is shaped by several factors, including its latitude, topography, ocean currents, and monsoons (Figure 2). According to Köppen’s climate classification, Taiwan exhibits four climate types: a monsoon and trade-wind coastal climate in the south, a mild, humid climate in the north, a wet–dry tropical climate in the west, and a temperate rainy climate with a dry winter in the mountain areas [23].

**Figure 3:**
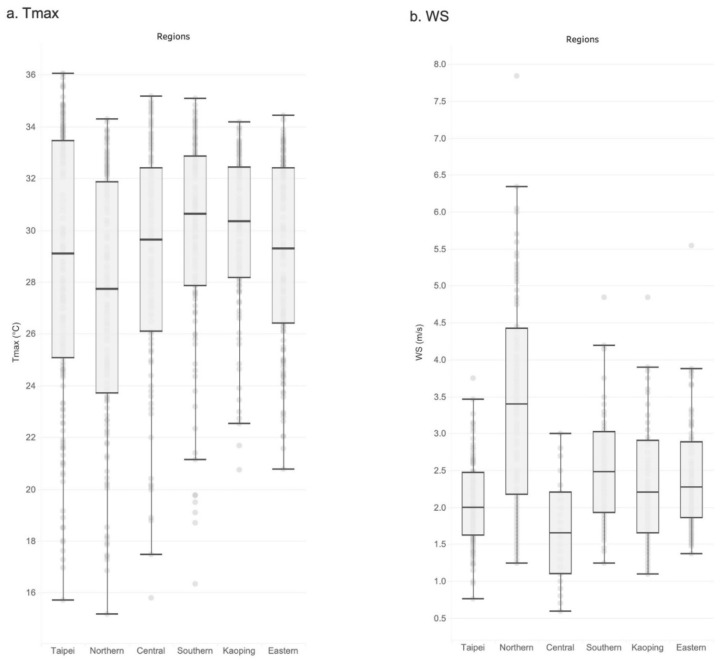
Box plot of the (**a**) maximum daily temperature (Tmax) and (**b**) wind speed (WS) in the six regions (Regions a–f), which illustrates that despite its small size, Taiwan’s varied topography still leads to six regions (Regions a–f) exhibiting distinctly different climatic characteristics.

Our study divided Taiwan into six regions: Taipei (Region a: station 1–6), Northern (Region b: station 7–8), Central (Region c: station 9), Southern (Region d: station 10–11), Kaoping (Region e: station 12–13), and Eastern regions (Region f: station 14–17). Details of the standard meteorological observation stations in each region are presented in Table 1. These divisions are based on Taiwan’s administrative divisions, which align with the divisions used by the Taiwan Centers for Disease Control, considering variations in local climate and population activities. These regions, with similar climatic environments and social circles about an hour apart, were analyzed for their potential impact on COVID-19 spread. Figure 3 displays the box plot of wind speed (WS) and maximum daily temperature (Tmax) across these six regions, highlighting the distinct climatic differences among them. This could potentially affect the transmission patterns of COVID-19.

### 2.2. Data Collection

#### 2.2.1. Collection of COVID-19 Case Data

Data on daily domestic COVID-19 cases were systematically gathered from the Taiwan Centers for Disease Control. This database, known for its comprehensive coverage and reliability, provides daily updates on domestic COVID-19 cases, thereby offering a robust source for tracking the pandemic’s progression in Taiwan. The data collection period, spanning from 1 March 2022 to 15 August 2022, was strategically chosen to encompass the peak of the COVID-19 outbreak in Taiwan. This specific timeframe was critical to capturing the dynamics of the pandemic during a significant wave of infections. Notably, 1 March 2022 marked a relatively low point in confirmed cases. Subsequently, there was a noticeable increase, with case numbers beginning to escalate, reaching double digits by 24 March and surpassing 100 by 31 March. By 15 August 2022, which falls in mid-August, the daily domestic COVID-19 cases had decreased to approximately 20,000, after which they stabilized for a period [4]. The dataset included the daily number of confirmed COVID-19 cases across each of the six regions (Regions a–f) in Taiwan. This regional breakdown enabled a detailed and nuanced analysis, reflecting the diverse epidemiological patterns across different parts of the country.

The peak periods of Taiwan’s pandemic, from March to August, aligned remarkably with the Fire-Qi Period, marked by unseasonal weather and temperatures often exceeding the yearly average. According to the Yunqi theory, a year is segmented into six phases, featuring two distinct “Fire-Qi Periods”—the minor ying and minor yang. Each of these periods lasts approximately two months, completing a full rotation every six years. Consequently, the Fire-Qi Periods exhibit a forward shift each year compared to the preceding one, a pattern meticulously detailed in Table 2. This synchronicity between Taiwan’s outbreak and the specific Fire-Qi Period pattern of that year, coupled with its potential connection to various weather factors, led us to focus our research on this specific period.

#### 2.2.2. Collection of Weather Data

Weather data for the same period (1 March to 15 August 2022) were obtained from the Central Weather Bureau of Taiwan. These data were meticulously compiled from multiple standard meteorological observation stations strategically located in each of the six regions. The weather parameters collected included:

- Diurnal temperature range (DTR): Calculated as the difference between the maximum and minimum daily temperatures, providing insight into daily temperature fluctuations.
- Maximum daily temperature (Tmax): Recorded in degrees Celsius (°C), representing the highest temperature reached each day. Tmax was found to have a positive correlation with the number of confirmed COVID-19 cases in prior studies.
- Relative humidity (RH): Expressed as a percentage, indicating the amount of moisture in the air. A prior study found no association between humidity and COVID-19 cases.
- Unease environmental condition factor (UECF): The unease environmental condition factor (UECF) was calculated for each region using the formula UECF = RH*Tmax/WS. This factor was included to assess the combined effect of temperature, humidity, and wind speed on environmental comfort and potential virus transmission [5].
- Wind speed (WS): Measured in meters per second (m/s), indicating the speed of wind observed at the stations. WS was found to have a positive correlation with the number of confirmed COVID-19 cases in prior studies [22,25,26].

To ensure a representative analysis of each region, we averaged each weather parameter across the standard meteorological stations of each region.

### 2.3. Data Analysis

Given the nonnormal distribution of the weather data such as temperature, RH, and WS, Spearman’s rank correlation test, a nonparametric method, was employed to determine the relationship between weather data and daily domestic COVID-19 cases in each region in Taiwan, which was also applied in other research [8,27,28,29].

#### Spearman’s Correlation Coefficients

The Spearman rank method, introduced by Spearman in 1904, offers a nonparametric (distribution-free) approach to measuring the correlation between two variables. Unlike methods that necessitate a linear relationship, Spearman’s method only requires that the association between variables be monotonically (steadily) increasing or decreasing. This search for a monotonic relationship is more inclusive and less restrictive compared to the pursuit of a linear correlation [30]. The Spearman’s Correlation Coefficients ($r_{s}$) can be calculated via the following equation:

$$r_{s}=1-6\;\frac{\Sigma d_{i}^{2}}{n\left(n^{2}-1\right)}$$

where $d_{i}$ is the difference between the ranks of corresponding variables, and n is the number of items or individuals being ranked. The value of $r_{s}\;$ranges from −1 to 1. A $r_{s}$ value close to 1 indicates a strong positive association, while a value close to −1 indicates a strong negative association. A value around 0 suggests no correlation.

**Figure 4:**
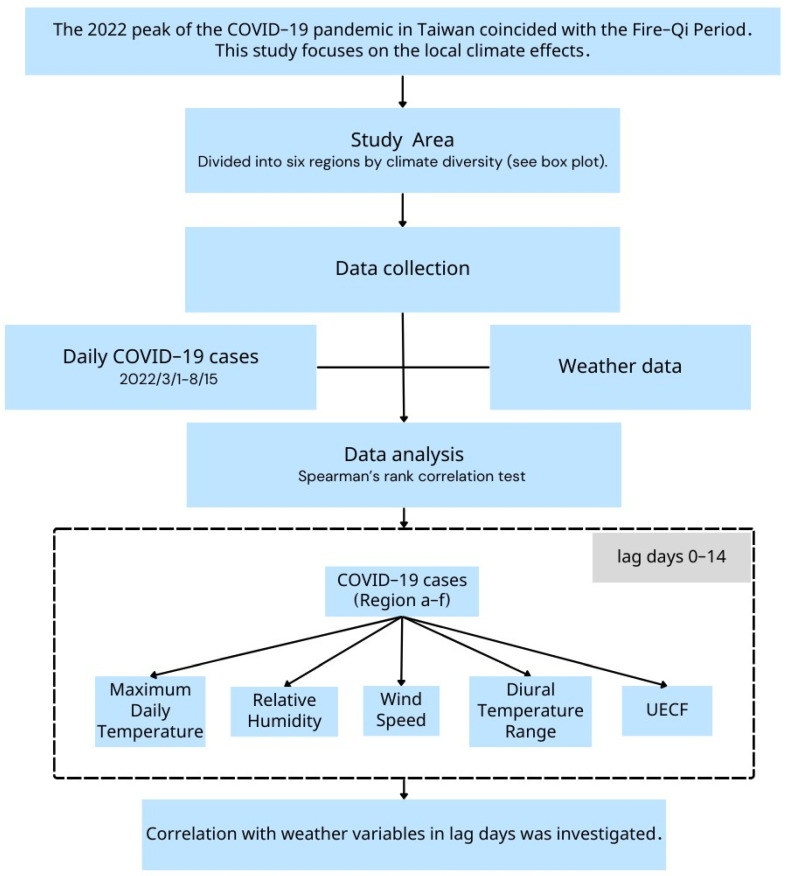
Research workflow details the methodology used to investigate the impact of local climate effects on COVID-19 transmission in Taiwan, focusing on the 2022 epidemic outbreak and its association with the Fire-Qi Period.

In our study, we calculated Spearman’s correlation coefficients to ascertain the strength and direction of the association between weather factors and COVID-19 case numbers. Additionally, our analysis took into account the potential lag effect of weather on COVID-19 transmission, incorporating a retrospective window of up to 14 days. Figure 4 illustrates the workflow of the study, detailing the methodology for investigating local climate effects on COVID-19 transmission in Taiwan.

## 3. Results

### 3.1. Daily Newly Confirmed Cases in Taiwan

Figure 1 displays the daily confirmed cases in Taiwan from 1 March to 15 August. On 31 March, the number of confirmed cases increased from tens to hundreds, and on 27 April, it surpassed ten thousand. The peak of the outbreak occurred on 26 May, with a record high of over 90,000 confirmed cases in a single day. The lower number of confirmed cases reported every seven days may be attributed to weekends. The reduced operational capacity of public health agencies and hospitals during weekends may have resulted in a decrease in the reported number of confirmed cases.

**Figure 5:**
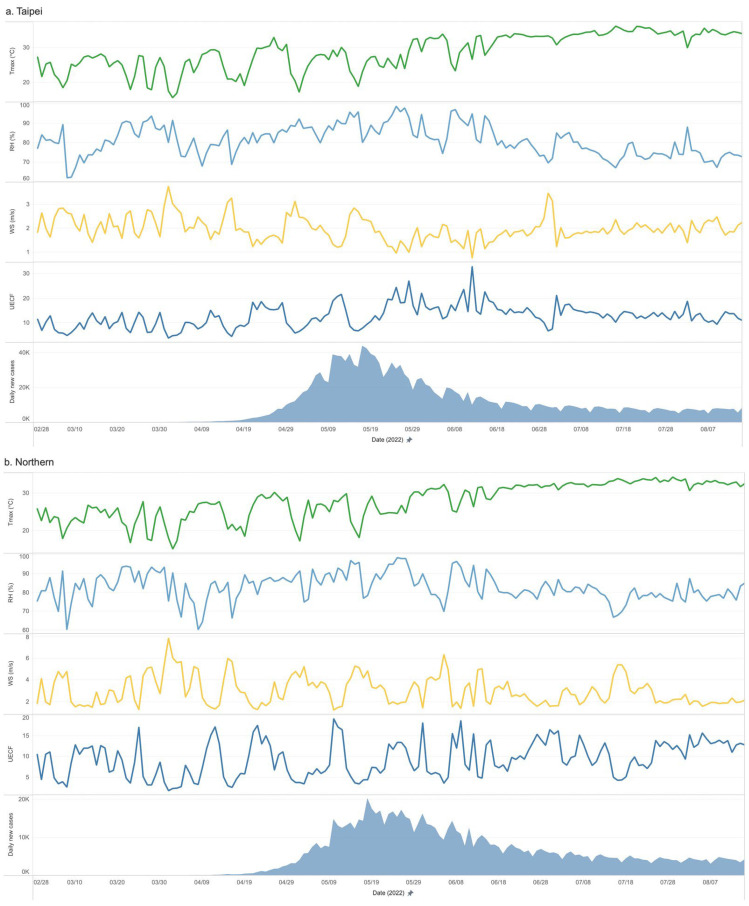

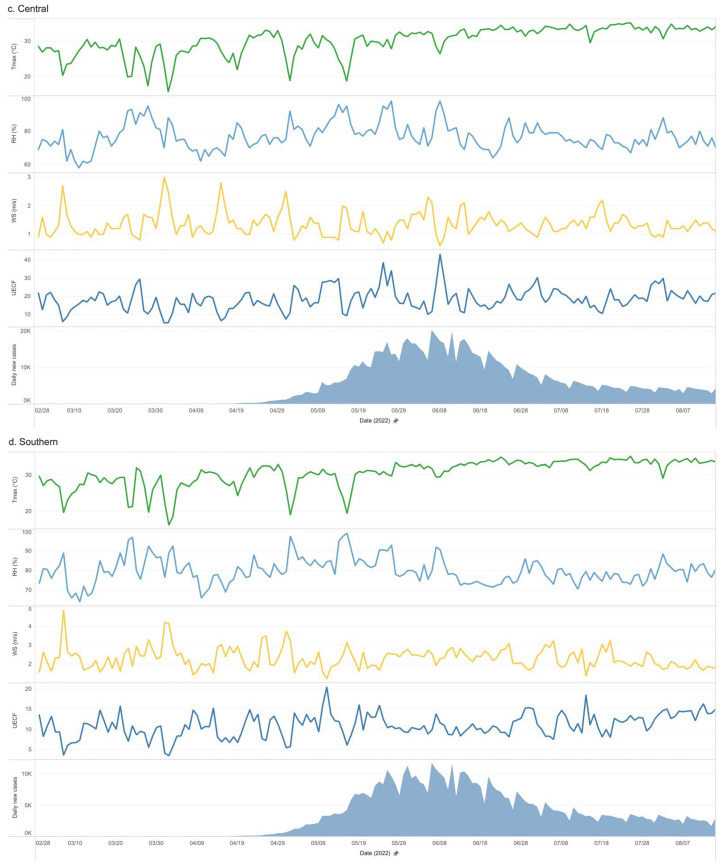

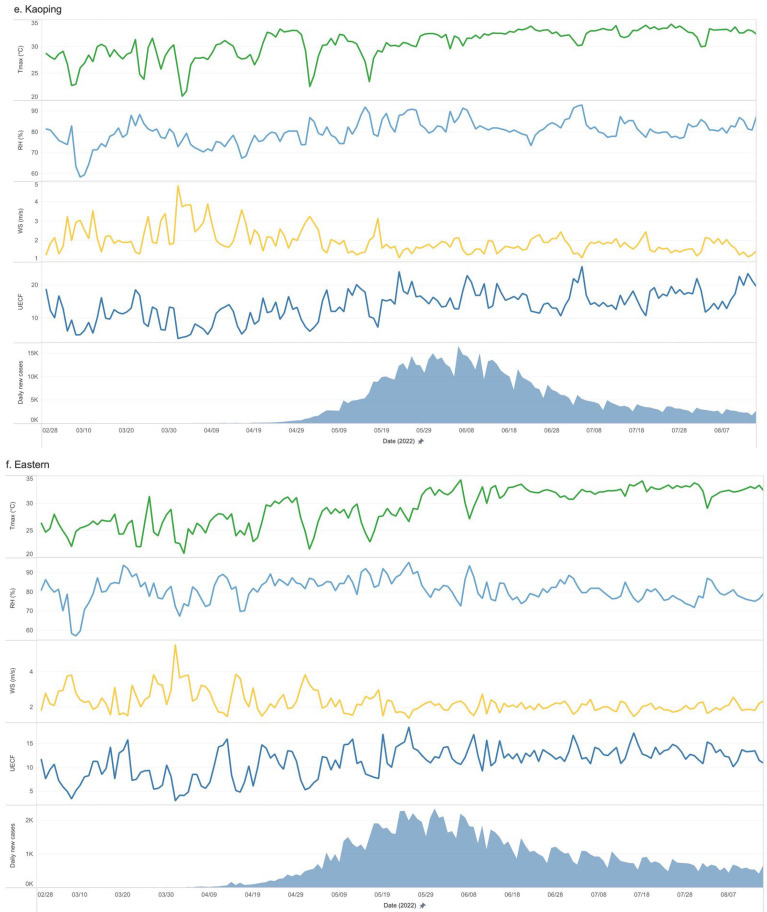
Relationship between weather variables (including Tmax, RH, WS, and UECF) and the number of confirmed cases in Regions a–f. From the chart, various weather data show different levels of fluctuations. Further statistical analysis to better understand the relationship between weather factors and case outbreaks will be discussed later.

The daily number of confirmed COVID-19 cases in each region was analyzed separately according to the potential local climate effects and population activities. Figure 5a–f depict the relationship between weather variables and the number of confirmed cases in each region.

### 3.2. Spearman’s Correlation Analysis

Table 3 presents the results of Spearman’s correlation analysis of the daily domestic confirmed cases and variable meteorological factors in each region of Taiwan.

**Figure 6:**
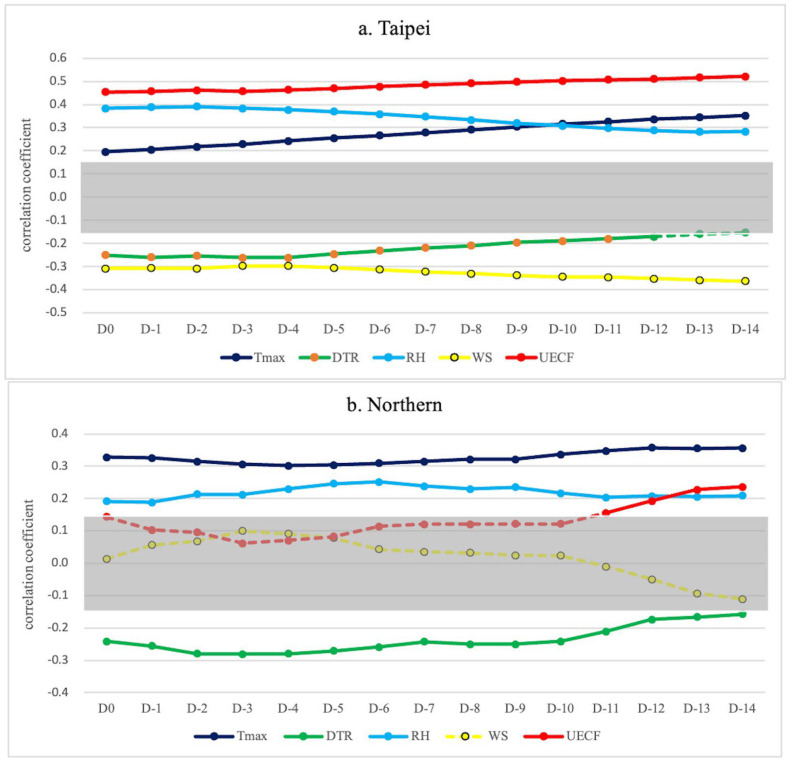

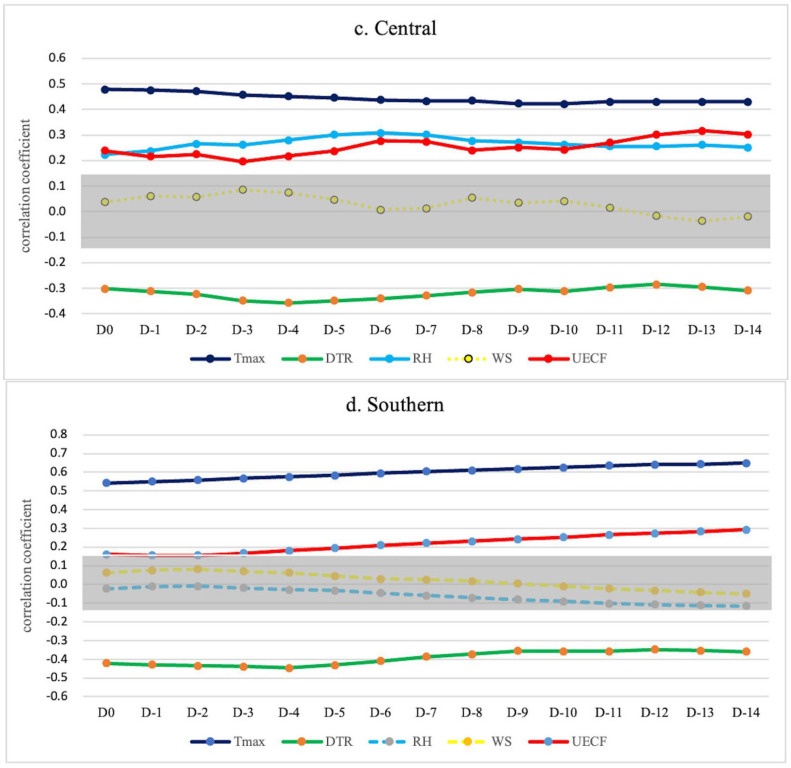

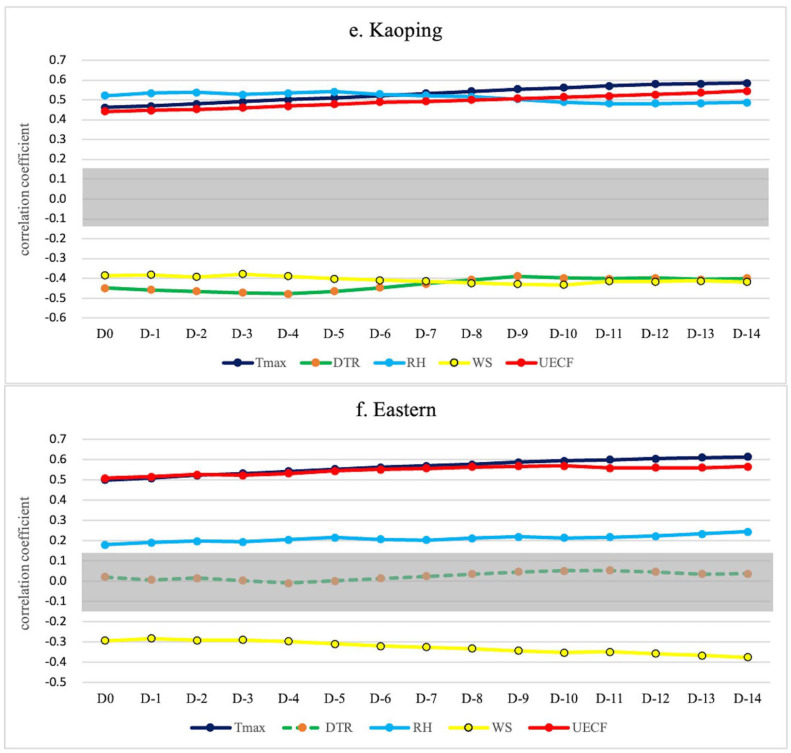
Correlation coefficient and strength of relationship for weather variables (including Tmax, RH, WS, and UECF) in lag days analysis (D-n = weather n days prior). The gray area indicates intervals where the correlation coefficient is less significant (ranging from 0.15 to −0.15), while the farther the weather variable data are from the gray area, the stronger the correlation. The weather variable data that are less significant are presented in the dashed line. The (a–f) represent Regions a–f, respectively, showing different strengths of relationship in weather variables such as Tmax, RH, and WS. However, when considering the CFC, such as the UECF, the strength of the correlation coefficient remains strong in almost every region.

In addition to the 1–14-day incubation period of SARS-CoV-2, the lag effect of weather on COVID-19 was considered. The data included confirmed cases, and weather variables were traced back 14 days for analysis (D0 = weather on the day, D-1 = weather one day prior, and D-n = weather n days prior). Figure 6 a–f are graphs of the data presented in Table 3. The gray area indicates intervals where the correlation coefficient is less significant (ranging from 0.15 to −0.15). Values that exceed the gray area show a significant correlation.

The correlation between new daily domestic COVID-19 cases and various weather factors within a 14-day lag was investigated in each region of Taiwan. The results showed a significant positive correlation between daily cases and Tmax. RH was also significantly correlated with new cases in Regions a, b, c, e, and f, while WS was significantly negatively correlated with new cases in Regions a, e, and f. Furthermore, the DTR was significantly negatively correlated with new cases in Regions a, b, c, d, and e.

This study also examined the correlation between new cases and the UECF (RH*Tmax/WS), which was found to be significantly correlated with new cases in Regions a, c, d, e, and f. In Region b, a positive correlation was observed during D11–D14.

## 4. Discussion

Numerous studies have been conducted on the impact of weather variables on the COVID-19 pandemic since the pandemic began in 2019, and heterogeneous results have been reported. While a great number of regional studies from different countries have shown an association between weather conditions and the COVID-19 pandemic [7,8,9,10,11], a large study review claimed the opposite [6]. Given the dynamic relationship among the environment, viruses, and populations, our study divided Taiwan into six regions to closely examine these dynamics. A prior study [22] identified a significant weather-related impact on COVID-19 in Taiwan, particularly in areas close to meteorological stations, which are close to the outbreaks of COVID-19. Our current research further explores this connection across these regions, considering the varying incubation periods of different COVID-19 variants [31] and the potential lag effects of weather, with data analysis extending 14 days prior to case confirmation. Figure 5a–f show the relationship between weather factors and the number of confirmed cases in each region.

In addition, both different incubation periods for different variants of COVID-19 [31] and the lag effect of weather on the incidence of COVID-19 have been proposed [32]. The collected data were traced back 14 days for analysis.

### 4.1. Meteorological Factors and COVID-19 Incidence

Although the prevailing literature generally posits that higher temperatures have a detrimental effect on COVID-19 transmission, leading to fewer confirmed cases [33,34,35], our findings present a different perspective. Our study identified a significant positive correlation between Tmax and confirmed COVID-19 cases across Taiwan, aligning with prior findings [7,8,15,22,36]. Despite evidence that SARS-CoV-2 loses viability at higher temperatures [37], the increase in cases suggests an impactful role of temperature in COVID-19 spread.

Contrary to earlier findings [6,22,38], which showed no link between RH and COVID-19, our analysis across Regions a, b, c, e, and f revealed a positive correlation, in line with more recent findings [8,9,15,39,40]. However, other studies have indicated a negative correlation between humidity and confirmed cases [10,41]. The positive correlation observed in our study could be due to the longer study period and broader area covered, with higher humidity possibly driving people indoors and increasing infection risks.

Our study showed a negative correlation between WS and newly confirmed COVID-19 cases in Regions a, e, and f, aligning with earlier research [8,17,38,39,42,43] and supporting the hypothesis that higher wind speeds might dilute airborne virus particles, reducing transmission [44].

Additionally, our findings indicated a significant negative correlation between the DTR and COVID-19 cases in Regions a, b, c, d, and e, consistent with studies suggesting that a narrower DTR might extend the virus’s viability [41,45].

### 4.2. Unease Environmental Condition Factor (UECF)

The concept of the unease environmental condition factor (UECF) was first proposed in a prior study [22], with the characteristics of increased temperature and relative humidity and decreased wind speed; this factor makes people feel uncomfortable and more likely to become sick. Our study revealed a significant positive correlation with daily confirmed cases in Regions a, c, d, e, and f, while a positive correlation was observed on lag days 11 to 14 in Region b. Region b includes the Xinwu and Hsinchu standard observation stations. The wind in Xinwu blows mainly from the sea, and the wind speed is higher than that at other stations, which may delay the weather impact.

Figure 6a–f describe the correlation between each meteorological factor and COVID-19 incidence with lag days 1 to 14. Analyzing weather factors individually reveals that correlations with outbreak patterns differ across countries. We propose that these factors interact. Considering all meteorological factors together, as in the unease environmental condition factor (UECF), is essential for a clearer understanding of weather’s impact on disease transmission. In addition, the Tmax remained the most relevant factor, aligning with the concept of “Fire-Qi” in traditional Chinese medicine and supporting findings on infectious disease epidemics in a prior study [22], and maybe the main weather factor in this COVID-19 outbreak. Since the complex interaction between weather factors and the risk of infectious diseases has been investigated, such as hand, foot, and mouth disease [46,47,48], MERS-COV [49,50], dengue [51,52,53], and influenza [54,55], we considered that the dynamic relationship between weather factors such as Tmax, RH, and WS can change environmental comfort, ultimately affecting human activities and the potential for COVID-19 transmission.

### 4.3. Local Climate Effect and Cyclic Climatic Variation

Numerous regional studies from different countries have shown the association between weather conditions and the COVID-19 pandemic, but conflicting results with positive and negative correlations were found [7,8,9,10]. As we mentioned above, the interaction of different weather factors and their combined impact on the environment and the human body should be considered. Moreover, the selection of local meteorological factors is a critical point. With the development of industrial society, the local climate not only affects environmental comfort but also affects people’s tendency to engage in activities [56], which is one of the main factors in the spread of the virus. Those reasons may explain why a large study review showed no association between weather conditions and the COVID-19 outbreak [6]. When diverse weather patterns across regions are considered together, the overall impact of weather factors on COVID-19 transmission appears to be reduced.

In particular, most studies conducted on the impact of weather variables on the COVID-19 pandemic indicated that the peak of the COVID-19 pandemic occurred in autumn or winter [57,58], but in reality, several peaks of COVID-19 in spring or summer have been observed worldwide since 2020. In addition to disease control policies such as vaccination coverage and quarantine control measures, cyclic climatic variations may lead to specific weather conditions that affect infectious disease epidemics.

**Figure 7:**
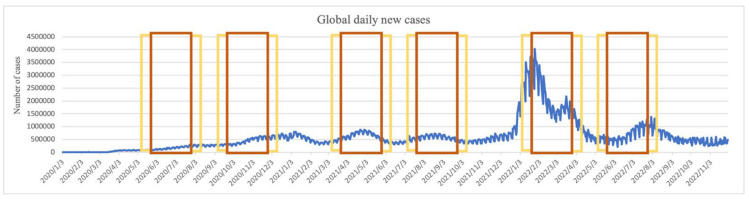
Global daily new cases matched the Fire-Qi Period from 2020 to 2022. Most of the peak periods of the pandemic coincided with the Fire-Qi Period (the red box). The yellow boxes represent the 30 days before and after the Fire-Qi Period, which was the deviation range in Yunqi theory.

The “Fire-Qi Period,” based on the Yunqi theory of Chinese medicine in Huangdi’s Internal Classic (Huang Di Nei Jing), is a certain period of the year that is prone to infectious disease epidemics, with the characteristics of unseasonal weather and possibly higher temperatures than the annual average temperature. There are two “Fire-Qi Periods” in a year (known as the minor ying and minor yang) [59], each lasting approximately two months, with a regular rule of two months in advance every year and repeating every six years, with each year forward shift compared to the previous one. This means that each year, the Fire-Qi Periods occur slightly earlier than in the previous year, as detailed in Table 2. Through a comparison of the COVID-19 pandemic from 2020 to the present, we observed that most of the peak periods of the pandemic coincided with the Fire-Qi Period (Figure 7). We proposed that the Fire-Qi Period represents a specific weather condition and that such cyclic climatic variations can affect infectious disease epidemics and can even be observed globally. The concept of the UECF as components of the Climate Factor Complex (CFC) was based on actual observations and attempted to explain the specific weather conditions of the Fire-Qi Period, which may serve as a methodological model for global application.

In summary, the impact of weather conditions on the COVID-19 pandemic was more clearly reflected when considering local climate effects. Second, meteorological factors should not be analyzed alone in relation to COVID-19 incidence. We consider it important that weather conditions change environmental comfort and make people more susceptible to illness. The UECF, considering the interaction of weather factors, could reveal a correlation with the COVID-19 pandemic. Third, specific weather conditions under cyclic climatic variation that affect infectious disease epidemics may be one of the warning signals that indicate the upcoming peak of a pandemic, guiding the implementation of health policies and sufficient supply preparations.

## 5. Conclusions

This study focused on the relationship between local weather conditions and COVID-19 transmission in six regions of Taiwan. The results showed that Tmax and RH were positively correlated with daily confirmed COVID-19 cases, while WS and DTR were negatively correlated. Furthermore, the UECF showed a significant positive correlation with COVID-19 incidence. The impact of weather conditions on the COVID-19 pandemic was revealed more clearly when considering local climate effects and the interaction of weather factors, even with high vaccination coverage. Furthermore, the cyclic climatic variation that affects infectious disease epidemics could be observed globally. The Fire-Qi Period, representing the concept of a specific weather condition affecting environmental comfort and human susceptibility to infection, was proposed.

Although our study was conducted only in Taiwan, this methodological model established by the Climate Factor Complex (CFC) could be widely applicable. The CFC integrates several climate factors, including temperature, humidity, rainfall, wind speed, sunshine, and air pressure, which collectively can have a large-scale impact on the environment [26]. From our conclusions, when climatic conditions exhibit the following characteristics—higher temperatures and humidity, along with lower wind speed and DTR—it signals a red alert for epidemic transmission. Understanding this premise, when climatic conditions reach this epidemic red alert, can human intervention to adjust environmental conditions, such as reducing indoor temperature and humidity and enhancing air circulation, potentially reduce the incidence rate? These questions warrant further research and testing, hoping to more effectively provide early warnings for epidemics.

## Figures and Tables

**Table 1:** Latitude and longitude for standard observation stations across six regions (Regions a–f) of Taiwan are shown, with each color representing a different region based on administrative divisions.

Region	Standard Observation Stations	Location		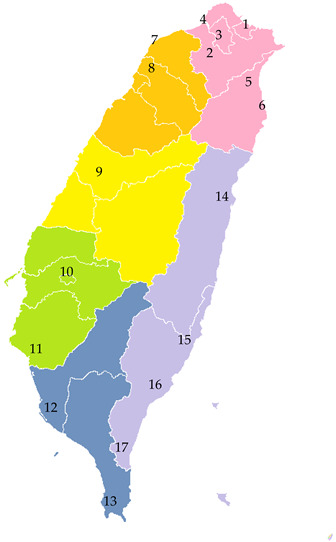
		north latitude	east longitude	
Taipei (Region a)	1. Keelung	25°1333′	121°7404′	
	2. Banqiao	24°9976′	121°4489′	
	3. Taipei	25°0376′	121°5148′	
	4. Tamsui	25°1648′	121°489′	
	5. Yilan	24°7639′	121°7565′	
	6. Su-ao	24°5967′	121°8573′	
Northern (Region b)	7. Xinwu	25°0067′	121°0474′	
	8. Hsinchu	24°8278′	121°0142′	
Central (Region c)	9. Taichung	24°1457′	120°6840′	
Southern (Region d)	10. Chiayi	23°4959′	120°4329′	
	11. Tainan	22°9932′	120°2047′	
Kaoping (Region e)	12. Kaohsiung	22°7304′	120°3125′	
	13. Hengchun	22°0038′	120°7463′	
Eastern (Region f)	14. Hualien	23°9751′	121°6132′	
	15. Chenggong	23°0974′	121°3734′	
	16. Taitung	22°7522′	121°1545′	
	17. Dawu	22°3556′	120°9037′	

**Table 2:** The Yunqi theory divides a year into six phases, including two “Fire-Qi Periods” (minor ying and minor yang). These periods repeat every six years, each lasting approximately two months.

Time Distribution of the Fire-Qi Period						
Years/Qi	1st Qi 1/19–21~3/20–22	2nd Qi 3/20–22~5/20–22	3rd Qi 5/20–22~7/22–24	4th Qi 7/22–24~9/22–24	5th Qi 9/22–24~11/21–23	6th Qi 11/21–23~1/19–21
2002, 2008, 2014, 2020, 2026, 2032, 2038, 2044, 2050	major yang	reverting ying	**minor ying**	major ying	**minor yang**	yang brightness
2003, 2009, 2015, 2021, 2027, 2033, 2039, 2045, 2051	reverting ying	**minor ying**	major yang	**minor yang**	yang brightness	major yang
2004, 2010, 2016, 2022, 2028, 2034, 2040, 2046, 2052	**minor ying**	major ying	**minor yang**	yang brightness	major yang	reverting ying
2005, 2011, 2017, 2023, 2029, 2035, 2041, 2047, 2053	major ying	**minor yang**	yang brightness	major yang	reverting ying	**minor ying**
2006, 2012, 2018, 2024, 2030, 2036, 2042, 2048, 2054	**minor yang**	yang brightness	major yang	reverting ying	**minor ying**	major yang
2007, 2013, 2019, 2025, 2031, 2037, 2043, 2049, 2055	yang brightness	greater yang	reverting ying	**minor ying**	greater ying	**minor yang**

The terms minor ying (major fire), minor yang (minor fire), and major yang likely represent heat and cold; major ying and yang brightness could indicate dampness or dryness; and reverting ying may signify wind.

**Table 3:** Spearman’s correlation analysis of the daily domestic confirmed cases and variable meteorological factors was traced back 14 days in each region of Taiwan.

Region		D0	D-1	D-2	D-3	D-4	D-5	D-6	D-7	D-8	D-9	D-10	D-11	D-12	D-13	D-14
Tmax	a	0.196 *	0.205 **	0.218 **	0.229 **	0.242 **	0.255 **	0.267 **	0.279 **	0.291 **	0.304 **	0.316 **	0.326 **	0.336 **	0.344 **	0.352 **
	b	0.328 **	0.325 **	0.314 **	0.306 **	0.302 **	0.304 **	0.309 **	0.314 **	0.321 **	0.321 **	0.336 **	0.347 **	0.357 **	0.355 **	0.356 **
	c	0.478 **	0.476 **	0.471 **	0.457 **	0.452 **	0.446 **	0.438 **	0.433 **	0.435 **	0.424 **	0.422 **	0.431 **	0.430 **	0.430 **	0.431 **
	d	0.542 **	0.550 **	0.559 **	0.568 **	0.576 **	0.584 **	0.595 **	0.604 **	0.611 **	0.619 **	0.626 **	0.635 **	0.641 **	0.644 **	0.649 **
	e	0.501 **	0.509 **	0.523 **	0.533 **	0.544 **	0.554 **	0.563 **	0.571 **	0.578 **	0.588 **	0.595 **	0.601 **	0.606 **	0.611 **	0.614 **
	f	0.462 **	0.469 **	0.480 **	0.491 **	0.501 **	0.511 **	0.522 **	0.532 **	0.543 **	0.553 **	0.562 **	0.571 **	0.579 **	0.581 **	0.584 **
DTR	a	−0.251 **	−0.260 **	−0.253 **	−0.262 **	−0.262 **	−0.246 **	−0.232 **	−0.219 **	−0.210 **	−0.196 *	−0.190 *	−0.180 *	−0.170 *	−0.159 *	−0.153 *
	b	−0.241 **	−0.256 **	−0.280 **	−0.281 **	−0.280 **	−0.271 **	−0.259 **	−0.242 **	−0.250 **	−0.250 **	−0.241 **	−0.211 **	−0.174 *	−0.166 *	−0.157 *
	c	−0.301 **	−0.312 **	−0.323 **	−0.348 **	−0.357 **	−0.348 **	−0.340 **	−0.328 **	−0.315 **	−0.303 **	−0.311 **	−0.296 **	−0.284 **	−0.294 **	−0.308 **
	d	−0.421 **	−0.429 **	−0.434 **	−0.439 **	−0.447 **	−0.430 **	−0.409 **	−0.387 **	−0.373 **	−0.355 **	−0.357 **	−0.357 **	−0.348 **	−0.354 **	−0.360 **
	e	0.023	0.008	0.016	0.004	−0.008	0.002	0.015	0.025	0.036	0.048	0.052	0.054	0.048	0.037	0.039
	f	−0.448 **	−0.458 **	−0.465 **	−0.472 **	−0.478 **	−0.465 **	−0.447 **	−0.426 **	−0.407 **	−0.389 **	−0.397 **	−0.401 **	−0.398 **	−0.406 **	−0.399 **
RH	a	0.383 **	0.388 **	0.391 **	0.384 **	0.378 **	0.370 **	0.359 **	0.347 **	0.334 **	0.320 **	0.309 **	0.298 **	0.288 **	0.282 **	0.283 **
	b	0.191*	0.188*	0.213 **	0.212 **	0.229 **	0.246 **	0.251 **	0.238 **	0.229 **	0.235 **	0.216 **	0.203 **	0.208 **	0.206 **	0.209 **
	c	0.224 **	0.238 **	0.266 **	0.262 **	0.280 **	0.301 **	0.308 **	0.302 **	0.277 **	0.272 **	0.263 **	0.256 **	0.256 **	0.261 **	0.252 **
	d	−0.023	−0.010	−0.009	−0.019	−0.028	−0.033	−0.046	−0.060	−0.070	−0.080	−0.090	−0.101	−0.108	−0.111	−0.116
	e	0.181*	0.192*	0.198*	0.195*	0.206 **	0.217 **	0.207 **	0.204 **	0.214 **	0.221 **	0.215 **	0.218 **	0.223 **	0.234 **	0.246 **
	f	0.522 **	0.534 **	0.537 **	0.526 **	0.534 **	0.542 **	0.528 **	0.521 **	0.515 **	0.504 **	0.489 **	0.481 **	0.481 **	0.484 **	0.486 **
WS	a	−0.309 **	−0.307 **	−0.309 **	−0.297 **	−0.297 **	−0.305 **	−0.313 **	−0.323 **	−0.330 **	−0.338 **	−0.345 **	−0.346 **	−0.352 **	−0.358 **	−0.363 **
	b	0.014	0.056	0.068	0.100	0.091	0.078	0.043	0.036	0.032	0.025	0.024	−0.010	−0.050	−0.093	−0.111
	c	0.038	0.061	0.058	0.086	0.075	0.048	0.008	0.013	0.054	0.035	0.042	0.016	−0.016	−0.036	−0.019
	d	0.063	0.076	0.083	0.071	0.062	0.046	0.030	0.025	0.019	0.005	−0.008	−0.023	−0.032	−0.042	−0.049
	e	−0.292 **	−0.282 **	−0.290 **	−0.288 **	−0.296 **	−0.308 **	−0.319 **	−0.325 **	−0.332 **	−0.342 **	−0.351 **	−0.348 **	−0.356 **	−0.366 **	−0.374 **
	f	−0.385 **	−0.382 **	−0.393 **	−0.378 **	−0.389 **	−0.402 **	−0.409 **	−0.415 **	−0.424 **	−0.428 **	−0.433 **	−0.414 **	−0.416 **	−0.413 **	−0.418 **
UECF	a	0.454 **	0.457 **	0.462 **	0.458 **	0.463 **	0.470 **	0.477 **	0.486 **	0.492 **	0.498 **	0.503 **	0.507 **	0.511 **	0.516 **	0.522 **
	b	0.143	0.103	0.095	0.062	0.070	0.082	0.114	0.120	0.120	0.122	0.122	0.155*	0.192*	0.227 **	0.236 **
	c	0.239 **	0.216 **	0.225 **	0.197 *	0.217 **	0.238 **	0.277 **	0.275 **	0.241 **	0.251 **	0.243 **	0.270 **	0.301 **	0.317 **	0.303 **
	d	0.162 *	0.155 *	0.155 *	0.168 *	0.181 *	0.195 *	0.209 **	0.221 **	0.231 **	0.243 **	0.253 **	0.266 **	0.274 **	0.283 **	0.293 **
	e	0.442 **	0.446 **	0.452 **	0.459 **	0.468 **	0.478 **	0.488 **	0.493 **	0.499 **	0.507 **	0.514 **	0.520 **	0.527 **	0.536 **	0.545 **
	f	0.510 **	0.517 **	0.528 **	0.523 **	0.533 **	0.545 **	0.552 **	0.558 **	0.564 **	0.568 **	0.570 **	0.560 **	0.561 **	0.562 **	0.567 **

* Correlation is significant at the 0.05 level; ** correlation is significant at the 0.01 level; Tmax: maximum temperature; DTR: diurnal temperature range; RH: relative humidity; WS: wind speed; UECF: unease environmental condition factor.

## Data Availability

Data are contained within the article.
